# Prognostic Value of Massiveness Parameters Measured on Baseline FDG PET in Advanced‐Stage Hodgkin Lymphoma

**DOI:** 10.1002/cam4.71462

**Published:** 2025-12-13

**Authors:** S. Draye‐Carbonnier, S.‐D. Mihailescu, P. Pinochet, E. Texte, A. Stamatoullas‐Bastard, P. Vera, S. Becker, P. Decazes

**Affiliations:** ^1^ Department of Nuclear Medicine Centre Henri Becquerel Rouen France; ^2^ Department of Statistics and Clinical Research Unit Centre Henri Becquerel Rouen France; ^3^ Department of Hematology Centre Henri Becquerel Rouen France; ^4^ QuantIF‐LITIS (EA 4108‐FR CNRS 3638), Faculty of Medicine University of Rouen Rouen France

**Keywords:** ^18^F‐FDG PET/CT, Hodgkin lymphoma, outcome prediction, prognosis, radiomics

## Abstract

**Purpose:**

The prognostic value of radiomic quantitative features measured on pretreatment ^18^F‐FDG PET/CT was investigated in patients with advanced‐stage Hodgkin lymphoma (HL).

**Methods:**

We conducted a retrospective study of 176 HL patients diagnosed between 2006 and 2017. A dozen of PET/CT‐derived features were extracted via *Oncometer3D* from baseline ^18^F‐FDG PET/CT images. The receiver operating characteristic (ROC) curves, Kaplan–Meier method, and Cox analyses were used to assess the prognostic factors for Overall Survival (OS) and Progression‐Free Survival (PFS) censored at 5 years.

**Results:**

Four different clusters were identified among the 12 PET parameters analyzed: activity, tumor burden, fragmentation‐massiveness, and dispersion. On ROC analyses, medEdgeD, a massiveness parameter, had the highest AUC for OS (0.72) and PFS (0.6). Patients with high baseline medEdgeD had a significantly worse PFS (*p* = 0.04) and OS (*p* = 0.003) in both Kaplan–Meier and Cox univariate analyses. Furthermore, medEdgeD remained statistically significant in a multivariate analysis (*p* = 0.008 for OS and *p* = 0.014 for PFS) including various TEP and clinical parameters used in daily routine. In addition, in sub‐group analyses, a significantly worse prognosis was observed for patients with ABVD and with high baseline medEdgeD value (*p* = 0.0082 for OS and *p* = 0.001 for PFS). Moreover, in the bulky subgroup, medEdgeD improved prognostic accuracy (*p* = 0.016).

**Conclusion:**

PET parameters describing massiveness, in particular medEdgeD, are significantly correlated with prognosis in HL patients for OS and PFS, especially when treated with ABVD.

## 1 Introduction

Classical Hodgkin Lymphoma (HL), a B‐cell malignancy characterized by the presence of Hodgkin and Reed‐Sternberg cells, accounts for 10% of all newly diagnosed lymphomas but is the most common lymphoid malignant disease in young adults [1, 2].

HL is associated with a good prognosis with an estimated 5‐year OS of 86.6% [3]. Most of the patients respond to the standard first‐line treatment and are cured. However, in selected cases, the disease relapses, mostly within 3 years or remains primarily refractory.

^18^F‐fluoro‐2‐deoxy‐D‐glucose positron emission tomography/computed tomography (^18^F‐FDG PET/CT) has become an integral part of modern management of HL patients [4, 5] and is nowadays routinely recommended for pretreatment staging, interim, and end‐of‐therapy assessment [6, 7, 8], using the Deauville 5‐point scale (D5S) by quantifying the residual FDG uptake in pathological sites with two reference organs (mediastinal blood pool and liver) [9]. Furthermore, ^18^F‐FDG PET/CT is more accurate than conventional methods such as Contrast‐Enhanced Computed Tomography (CE‐CT) scan or bone marrow biopsy in the staging of the disease [10, 11].

Quantitative PET is an area of growing interest and appears to be more reproducible and more accurate for response or prognosis assessment. A variety of imaging‐derived quantitative parameters have recently been reported with the development of radiomics. In addition to TMTV, which assesses tumor burden, describing both the tumor size, the activity of tumor and microenvironment cells, and has been shown to be an independent prognostic factor in localized LH [12, 13, 14, 15], more complex quantitative parameters can be extracted from ^18^F‐FDG PET/CT data and analyzed [16]. These radiomic parameters can, for example, represent the tumors' massiveness‐fragmentation (TVSR; nROI; itErosion; medEDGE; medPCD), dispersion (*D*_max_; TumBB), or activity (SUVmax; SUVmean and TLG) [17]. At present time, none have yet been translated into daily‐routine clinical practice [18].

HL patients with a high risk of treatment failure or early relapse cannot be easily identified by classical prognostic clinical indicators such as the Hasenclever International Prognostic Score (IPS) [19]. Thus, there is a need for new reliable prognostic factors to better select high‐risk patient categories that can benefit from personalized, risk‐adapted treatment strategies shortly after diagnosis.

The aim of the present study was to measure ^18^F‐FDG PET/CT‐derived quantitative parameters in patients newly diagnosed with HL and investigate their potential role as predictive factors at baseline imaging.

## 2 Materials and Methods

### 2.1 Patients

We carried out a retrospective observational monocentric study in the Nuclear Medicine department of Henri Becquerel Cancer Centre, Rouen, France. The study was approved by the Institutional Review Board (no. 2102B). Patients were informed about the use of anonymized data for research and their right to oppose this use. Due to the retrospective nature of the study, the requirement for obtaining informed consent was waived by the IRB of the Henri Becquerel Center Internal Ethics Committee. The study respected the ethical principles of the 2008 Helsinki Declaration.

The inclusion criteria were as follows: (1) patients were 16 years old or above with a (2) histologically proven HL and (3) advanced stage Ann Arbor based on the German Hodgkin Study Group (GHSG) criteria, which include stage IIb with bulky disease and stages III–IV confirmed by the hematology local committee, (4) treated in first line with 6 or 8 cycles of ABVD regimen (Adriamycin, Bleomycin, Vinblastine, and Dacarbazine) or 2–6 cycles of BEACOPP regimen (Bleomycin, Etoposide, Adriamycin, Cyclophosphamide, Vincristine, Procarbazine, and Prednisone) [5], with a mandatory baseline ^18^F‐FDG PET/CT examination, performed prior to the initiation of therapy between March 2006 and December 2017 and [6] the patient's nonopposition statement.

Exclusion criteria were as follows: (1) patients with nodular lymphocyte predominant lymphoma, (2) other concomitant diseases with ^18^F‐FDG avidity, and (3) no available ^18^F‐FDG PET/CT at baseline.

Fifteen patients initially treated with BEACOPP and later de‐escalated to ABVD were classified within the BEACOPP group according to the intent‐to‐treat principle. De‐escalation was guided by interim PET response assessment after two cycles, following local protocols and guidelines at the time.

### 2.2 Clinical Data

Baseline patient's characteristics and survival data were obtained from internal medical records. Clinical data on the following variables were obtained from all the patients: age at disease onset, gender, Eastern Cooperative Oncology Group (ECOG) performance status, presence of B symptoms, treatment regimen, and disease characteristics: Ann Arbor stage, mediastinal‐thoracic index (bulk if MTI > 0.35), and results of bone marrow biopsy, as performed at this time in line with national standards, especially in cases with systemic symptoms or unexplained cytopenias, to detect stage IV disease. Current guidelines have since refined indications for its use.

The IPS score was also calculated for each patient. It is defined as the number of adverse prognostic factors including age, gender, serum albumin level, hemoglobin level, Ann Arbor stage, leukocytosis, and lymphocytopenia [19].

The datasets used and/or analyzed during the current study are available from the corresponding author on reasonable request.

### 2.3 PET Acquisition and Interpretation

All patients underwent ^18^F‐FDG PET/CT with acquisitions performed according to the Society of Nuclear Medicine and Molecular Imaging (SNMMI) and the European Association of Nuclear Medicine (EANM) guidelines.

Patients were instructed to fast for at least 6 h before ^18^F‐FDG injection. Injection was not performed unless glucose blood level was below < 1.8 g/L. ^18^F‐FDG intravenous injected activity was around 3.5–4.5 MBq/kg, as a function of the PET/CT device used: *Biograph 16* (Siemens Medical Solutions, Malvern, PA, USA), *Biograph mCT 40* (Siemens Medical Solutions, Knoxville, TN, USA), or *Discovery 710* (GE Healthcare, Milwaukee, WI, USA) with a maximum activity of 450 MBq, after 30 min of rest. Scans were acquired approximately 60 min (±5 min) after injection.

CT scans for attenuation correction and anatomic localization were acquired from the mid‐thigh toward the base of the skull in most cases, and whole‐body acquisition was realized in others, with 100–120 kV and 120–150 mAs (based on the patient's weight), in helical mode. Contrast media injection was not performed.

Images were reconstructed with validated and commercially available iterative algorithms (Ordered‐Subset Expectation Maximization iterative reconstruction).

The PET systems were daily normalized, and the calibration coefficient was validated if the day‐to‐day variation remained below 0.3%. The global quantification, from the dose calibrator to the imaging system, was measured internally on a quarterly basis and double‐checked by the EARL's quality assurance program.

^18^F‐FDG PET/CT data was anonymized and collected in DICOM format. All the data was then retrospectively reviewed.

### 2.4 Segmentation

First, segmentation was performed automatically on fused PET/CT images using *AutoID* in *Syngo*.Via software (Siemens Healthineers). Hypermetabolic foci with SUV greater than 4.0 were segmented then automatically classified as pathological or physiological [18, 20, 21]. A visual check was performed by a trained nuclear physician (SDC), unaware of clinical outcomes or patient characteristics, to confirm inclusion of only pathological lesions. A manual verification and adaptation were then performed if needed.

### 2.5 Radiomic Parameters

A total of 12 quantitative 3‐D PET/CT‐derived parameters were then extracted with the software *Oncometer3D* (Figure S1):

- SUVmax, SUV (Standardized Uptake Value) is the activity concentration normalized to the injected activity, divided by the total body weight. SUVmax corresponds to the highest pixel's SUV in a volume of interest (VOI). The highest SUVmax over all lesions of the patient was reported.
- SUVmean, the mean value of SUV measured in all the tumors.
- TMTV, the tumor's metabolically active volume. It is the sum of the metabolic volumes of all nodal and extra‐nodal lesions and estimates the patient's total tumor burden.
- TLG, the total lesion glycolysis, obtained by multiplying the total metabolic activity of each lymphoma lesion (TMTV) by the SUVmean.
- TMTS, the total metabolic tumor surface, is the sum of the metabolic surfaces of all the tumors (tumor–healthy tissue interface).
- TVSR, the tumor volume surface ratio, is the ratio between the TMTV and the TMTS, describing the tumor fragmentation. A high value would indicate a massive tumor, while a low value would correspond to a more fragmented tumor.
- *D*_max_, the distance between the two lesions that are the furthest apart, is deduced from the 3D coordinates of the baseline TMTV and captures the dissemination/spread of the disease.
- TumBB is the volume of the bounding box including the tumors, representing the volume of tumor dispersion.
- nROI is the number of regions of interest, describing the number of unique tumors in the whole examination.
- itErosion, the iterative erosion, corresponding to the number of erosions necessitated to remove tumors from the images (pixels of 4 × 4 × 4 mm).
- medEdgeD, the median edge distance, corresponding to the median distance between the opposite edges of the tumors.
- medPCD, the median distance between the centroid of the tumors and their periphery, representing the tumors' massiveness.

### 2.6 Statistical Analysis

Statistical analysis was performed using the R software, version 4.0.4. Continuous variables are reported as mean ± standard deviation (SD) with minimal and maximal values. Categorical variables are expressed as numbers and percentages, and the median follow‐up was calculated using the reverse Kaplan–Meier method.

Progression‐Free Survival (PFS), a continuous variable, was defined from the date of diagnosis to progression, which was the first clinical suspicion of recurrence or diagnosis of recurrence on computed tomography (CT) or positron emission tomography (PET) or death of any cause. Overall Survival (OS) was estimated from the date of diagnosis to the date of death by any cause.

The relationship between the different PET metrics was characterized by the Spearman's rank correlation coefficient. Receiver operating characteristic (ROC) analyses for PFS and OS were used to determine the optimal cutoff value for each feature by maximizing the product of sensitivity and specificity. Sensitivity and specificity were calculated for that suitable cutoff. The area under the curve (AUC) was also calculated.

Survival curves for PFS/OS were obtained with the Kaplan–Meier method. The quantitative PET/CT variable was dichotomized according to the median of the values. For each binary variable, the comparison of survival curves between categories was assessed by the log‐rank test. The Cox Proportional Hazards Model was performed to evaluate the relationship between study variables and survival rates.

Statistical significance was set at a two‐tailed *p* value < 0.05.

## 3 Results

### 3.1 Patients' Characteristics and Outcomes

A total of 176 patients with previously untreated HL, considered as advanced stage according to Ann Arbor criteria and available baseline ^18^F‐FDG PET/CT, were included in the study.

Description of the population with clinical characteristics is available in Table 1. Patients (112 males and 64 females) had a median age of 37 years (range 17–85) and the majority (63.1%) received chemotherapy with the ABVD regimen.

**TABLE 1:** Baseline characteristics of Hodgkin lymphoma patients.

Characteristics	*n* = 176
**Age (years)**	
Mean (±SD)	39.7 (±15.5)
Median (25%; 75%)	37 (26; 49.5)
Min; max	17; 85
**Gender**	
Female	64 (36.4%)
Male	112 (63.6%)
**ECOG score**	
0–1	160 (92.0%)
2–4	14 (8.0%)
Unknown	2
**Binarized International Prognostic Score (IPS)**	
0–2	72 (41.6%)
3–7	101 (58.4%)
**Bulky disease**	
Yes	32 (18.2%)
No	144 (81.8%)
**Ann Arbor stage**	
IIb bulky	2 (1.1%)
III	72 (40.9%)
IV	102 (58.0%)
**B signs**	
Present	85 (48.3%)
Absent	91 (51.7%)
**Osteo‐medullary biopsy**	
Positive	19 (11.2%)
Negative	137 (80.6%)
Uninterpretable	5 (2.9%)
Not performed	9 (5.3%)
Unknown	6
**Treatment regimen**	
ABVD (standard, six cycles)	111 (63.1%)
BEACOPP (AHL 2011 protocol)	65 (36.9%)
Of which BEACOPP to ABVD de‐escalated	15 (8.5%)

The median follow‐up period was 5.42 years (4.96–6.03 years).

Mean values of the 12 baseline FDG PET/CT tumor features evaluated are reported in Table 2. Baseline median values of TMTV were 235 cm^3^ (mean 371.29 ± 429.72 cm^3^) and TLG was 1276 cm^3^ (mean 2512.94 ± 3351.59 cm^3^), similar to the data collected in the studies by Pinochet et al. [22] and Casasnovas et al. [23], measured with a different method of segmentation (e.g., threshold of 41% of the SUVmax).

**TABLE 2:** Description of the 12 parameters extracted from baseline PET/CT.

Parameters, median (25%; 75%), min; max	Study population (*n* = 176)
SUVmax	14.02 (11.37; 17.33) 5.5; 66.6
TMTV (cm^3^)	235 (101.52; 476.79) 4.48; 2596.86
TLG (g)	1276.16 (565.15; 3319.52) 19.84; 23909.78
*D*_max_ (mm)	516.42 (330.96; 655.51) 35.41; 943.46
SUVmean	5.85 (5.23; 6.64) 4.43; 12.39
TVSR (mm)	3.78 (3.05; 4.75) 2.15; 11.79
TMTS (cm^2^)	586.23 (253.12; 1035) 20.58; 3298.27
TumBB (cm^3^)	12,289.25 (5200.94; 23474.37) 15.36; 72038.4
nROI	22 (11–41) 1; 51
medEdgeD (mm)	25.6 (20.42; 34.26) 11.99; 96.18
medPCD (mm)	24.66 (16.85; 37.79) 6.93; 106.88
itErosion	1.27 (1.16; 1.48) 1.01; 3.25

### 3.2 FDG PET/CT Metrics and Correlations

**FIGURE 1:**
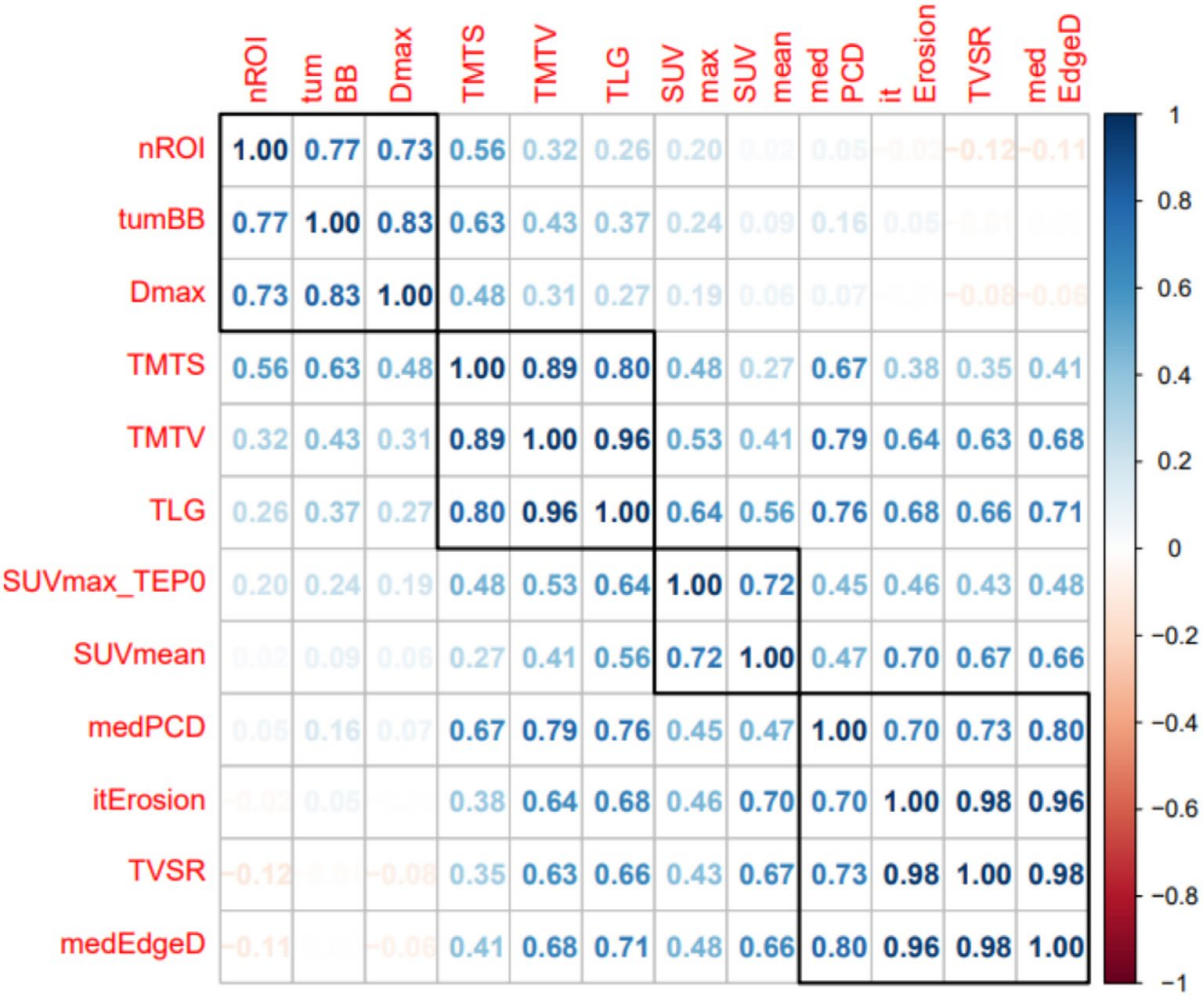
Correlogram between the 12 PET/CT parameters with numeric values. *D*_max_, largest distance between two lesions; itErosion, iterative erosion; medEdgeD, median edge distance; medPCD, median distance between the centroid of the tumor and its periphery; nROI, number of regions of interest; SUVmax, maximum standardized uptake value; SUVmean, mean standardized uptake value; TLG, total lesion glycolysis; TMTS, total metabolic tumor surface; TMTV, total metabolic tumor volume; TumBB, tumor bounding box; TVSR, tumor volume surface ratio.

As visible in the Spearman's correlogram (Figure 1), four different clusters, combining highly correlated parameters among the 12 PET parameters analyzed, could be identified:

- Activity (SUVmax; SUVmean),
- Tumor burden (TMTV; TMTS),
- Massiveness/fragmentation (medPCD; medEdgeD; itErosion; TVSR),
- Dispersion (*D*_max_; TumBB; nROI).

To note, TLG is by nature at the junction between activity and tumor burden.

### 3.3 ROC Curve Analysis

The ROC curve analysis for OS and PFS censored at 5 years is shown in respectively Tables 3 and 4 where optimal cutoff, AUC, sensitivity, and specificity are presented. Massiveness parameter medEdgeD had the highest area under the curve: AUC = 0.72; *p* = 0.0008, allowing a sensitivity of 60% and a specificity of 80.8% for OS and AUC = 0.60; *p* = 0.02 for PFS.

**TABLE 3:** Diagnostic performances of the 12 PET/CT‐derived parameters for OS using a ROC analysis.

	AUC	Cutoff value	Sensitivity (%)	Specificity (%)	*p*
SUVmax	**0.67**	15	75	60.9	**0.007**
TMTV	0.60	118	90	32.7	0.08
TLG	**0.62**	572	95	28.2	**0.04**
*D*_max_	0.45	149	100	6.4	0.78
SUVmean	**0.72**	6	75	73.1	**0.0007**
TMTS	0.54	359	80	37.8	0.3
TVSR	**0.71**	5	60	76.9	**0.001**
TumBB	0.47	34,128	15	92.9	0.68
nROI	0.40	50	5	98.1	0.92
medEdgeD	**0.72**	36	60	80.8	**0.0008**
medPCD	**0.69**	27	80	60.9	**0.003**
itErosion	**0.71**	1.32	75	62.8	**0.001**

*Note:* Values displayed in bold represent statistically significant results (*p* < 0.05).

**TABLE 4:** Diagnostic performances of the 12 PET/CT‐derived parameters for PFS using a ROC analysis.

	AUC	Cutoff value	Sensitivity (%)	Specificity (%)	*p*
SUVmax	0.53	14	62	51.6	0.26
TMTV	0.56	216	64	53.2	0.11
TLG	0.56	892	72	43.7	0.12
*D*_max_	0.47	743	10	95.2	0.73
SUVmean	0.55	6	46	69	0.16
TMTS	0.54	914	40	71.4	0.21
TVSR	**0.60**	5	44	75.4	**0.02**
TumBB	0.49	30,118	22	92.1	0.55
nROI	0.48	48	12	94.4	0.66
medEdgeD	**0.60**	26	62	61.1	**0.02**
medPCD	**0.59**	25	70	54	**0.03**
itErosion	**0.60**	1.29	64	59.5	**0.02**

*Note:* Values displayed in bold represent statistically significant results (*p* < 0.05).

Other massiveness/fragmentation parameters (itErosion, TVSR, and medPCD) had AUC significantly different from 0.5 for OS and PFS. Activity parameters, such as SUVmax, SUVmean, and partly TLG remained statistically significant only for OS prediction but not for PFS prediction. In contrast, dispersion parameters (*D*_max_, TumBB, and nROI) or burden parameters (TMTV, TMTS) had AUC no different from 0.5 for both OS and PFS predictions.

**FIGURE 2:**
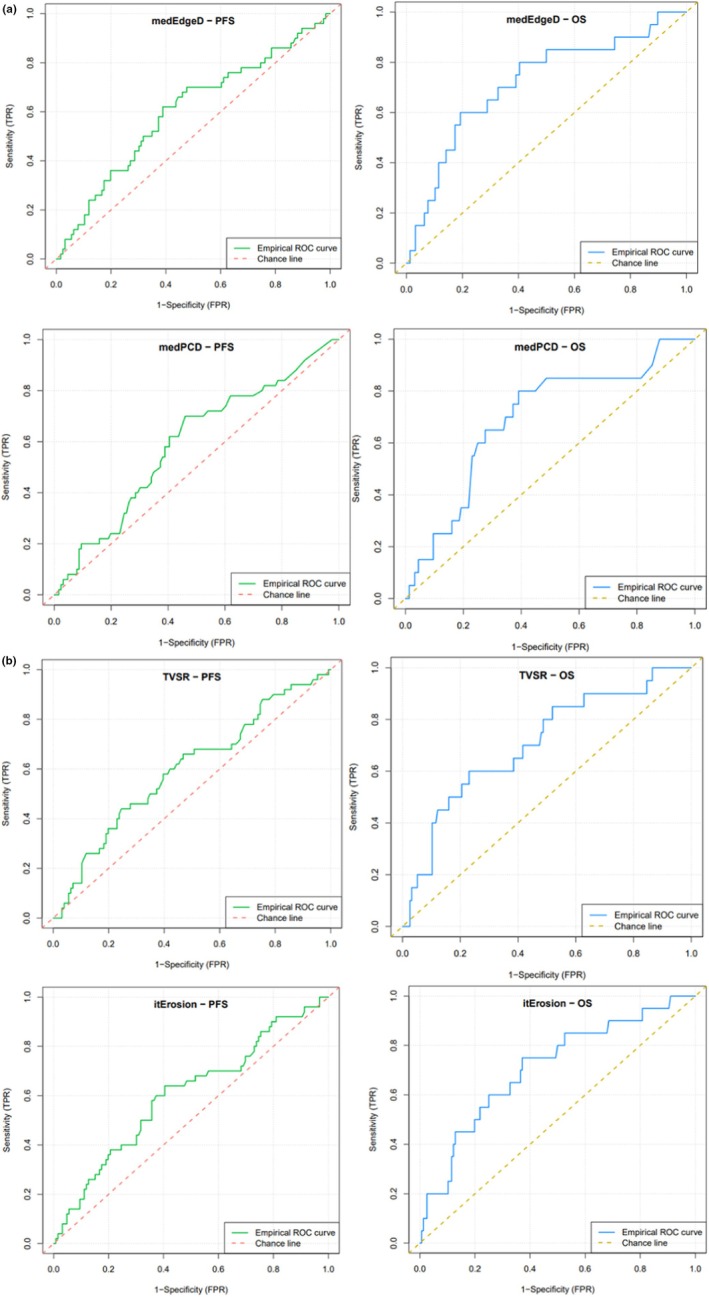
(a) ROC curves for PFS and OS censored at 5 years for fragmentation‐massiveness TEP parameters medEdgeD and medPCD. (b) ROC curves for PFS and OS censored at 5 years for fragmentation‐massiveness TEP parameters TVSR and itErosion.

Graphical representation of survival curves for OS and PFS for the four massiveness‐fragmentation parameters is available in Figure 2a,b.

### 3.4 Kaplan–Meier Survival Analysis

**FIGURE 3:**
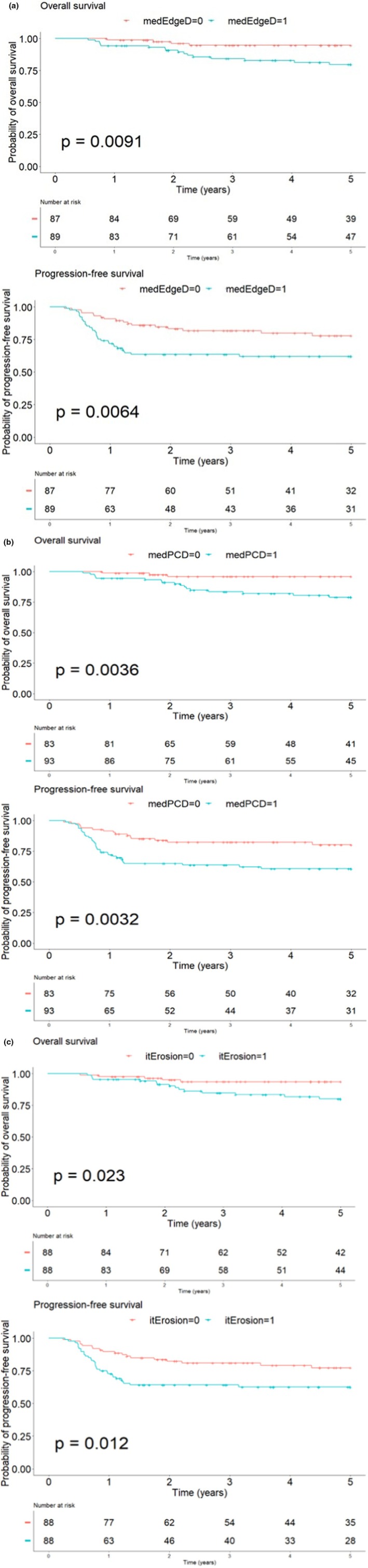
(a) Kaplan–Meier survival curves for OS and PFS according to medEdgeD. (b) Kaplan–Meier survival curves for OS and PFS according to medPCD. (c) Kaplan–Meier survival curves for OS and PFS according to itErosion.

A Kaplan–Meier survival analysis for OS and PFS at 5 years was performed according to the median values of the PET parameters. Massiveness parameters (medEdgeD, medPCD, and itErosion) had statistically significant log‐rank tests (all *p*‐values < 0.05). Graphical representations are presented in Figure 3a–c.

Only a tendency was shown for the fragmentation parameter TVSR (*p* = 0.067 for OS).

In addition, high baseline activity parameters (SUVmax and SUVmean) appeared to be linked to a worse prognosis for OS only.

### 3.5 Cox Univariate Analysis

Cox univariate analysis is presented in Tables 5 and 6. The univariate analysis showed that all high massiveness/fragmentation parameters measured (medEdgeD, medPCD, itErosion, and TVSR) were significantly associated with less favorable survival rates for OS (*p* < 0.05).

**TABLE 5:** Univariate Cox analysis for OS at 5 years.

Variables	HR	Lower bound 95% CI	Upper bound 95% CI	*p*
ECOG (≥ 2–4, ref.: 0–1)	2.85	0.83	9.84	0.10
IPS (≥ 3–7, ref.: 0–2)	1.34	0.53	3.41	0.54
Bulky (no, ref.: yes)	**0.34**	0.14	0.83	**0.02**
B signs (present, ref.: none)	**2.72**	1.05	0.83	**0.04**
Treatment (BEACOPP, ref.: ABVD)	0.37	0.11	1.27	0.12
SUVmax (≥ 14.02, ref.: < 14.02)	1.03	0.99	1.07	0.16
SUVmean (≥ 5.85, ref.: < 5.85)	**1.39**	1.11	1.73	**0.004**
TLG (≥ 1276.16, ref.: < 1276.16)	1.00	1.00	1.00	0.29
TVSR (≥ 3.78, ref.: < 3.78)	**1.30**	1.09	1.54	**0.003**
itErosion (≥ 1.27, ref.: < 1.27)	**3.35**	1.51	7.45	**0.003**
medPCD (≥ 24.66, ref.: < 24.66)	**1.02**	1.00	1.04	**0.03**
medEdgeD (≥ 25.6, ref.: < 25.6)	**1.03**	1.01	1.06	**0.003**

*Note:* Values displayed in bold represent statistically significant results (*p* < 0.05).

**TABLE 6:** Univariate Cox analysis for PFS at 5 years.

Variables	HR	Lower bound 95% CI	Upper bound 95% CI	*p*
ECOG (≥ 2–4, ref.: 0–1)	1.42	0.56	3.57	0.46
IPS (≥ 3–7, ref.: 0–2)	1.27	0.71	2.28	0.42
Bulky (no, ref.: yes)	0.71	0.37	1.40	0.32
B signs (present, ref.: none)	1.42	0.81	2.47	0.22
Treatment (BEACOPP, ref.: ABVD)	**0.37**	0.18	0.75	**0.006**
TVSR (≥ 3.78, ref.: < 3.78)	**1.14**	1.01	1.30	**0.04**
itErosion (≥ 1.27, ref.: < 1.27)	**2.02**	1.10	3.68	**0.023**
medPCD (≥ 24.66, ref.: < 24.66)	1.01	1.00	1.03	0.13
medEdgeD (≥ 25.6, ref.: < 25.6)	**1.02**	1.00	1.03	**0.04**

*Note:* Values displayed in bold represent statistically significant results (*p* < 0.05).

In contrast, neither classical clinical parameters such as IPS score, nor ECOG Performance Status were significantly associated with OS or PFS at 5 years (for OS, ECOG 2–4: *p* = 0.1; Index Prognostic 3–7: *p* = 0.54), while on the other hand, the presence of bulky criteria and B signs were significantly associated with worse OS at 5 years (B signs: *p* = 0.04; nonbulky: *p* = 0.02).

### 3.6 Multivariate Analysis

Due to the high correlation observed between some of the parameters with significant statistical value in univariate analysis, we chose to perform a multivariate stepwise analysis for OS and PFS combining clinical parameters, notably the bulky criteria and distinct PET parameters. MedEdgeD was the only parameter that retained statistical significance and independently predicted the 5‐year OS or PFS in the multivariate Cox regression model (Table 7a,b).

**TABLE 7:** Multivariate Cox Regression (stepwise backwards approach) including ECOG, IPS, bulky status, B signs, treatment regimen, SUVmean, medEdgeD, and TLG.

Variables	HR	Lower bound 95% CI	Upper bound 95% CI	*p*
(a) OS				
Bulky (no, ref.: yes)	0.47	0.19	1.20	0.11
B signs (present, ref.: none)	3.45	1.23	9.65	0.02
Treatment (BEACOPP, ref.: ABVD)	0.29	0.08	1.04	0.06
medEdgeD (≥ 25.6, ref.: < 25.6)	1.03	1.01	1.07	0.008
(b) PFS				
B signs (present, ref.: none)	1.69	0.95	3.00	0.07
Treatment (BEACOPP, ref.: ABVD)	0.33	0.16	0.69	0.003
medEdgeD (≥ 25.6, ref.: < 25.6)	1.02	1.004	1.04	0.014

The quantitative TEP parameter appeared to be statistically superior to the bulky criteria used in daily routine to evaluate prognosis.

### 3.7 Association of Parameters Treatment Regimen and medEdgeD

Patients could be divided into four sub‐groups: Association “1” for treatment by ABVD + low medEdgeD value (*n* = 53; 30%), “2” for treatment by ABVD + high medEdgeD value (*n* = 58; 33%), and “3” and “4” for respectively treatment by BEACOPP + low (*n* = 34; 19%) or high (*n* = 31; 18%) value of medEdgeD at baseline imaging.

**FIGURE 4:**
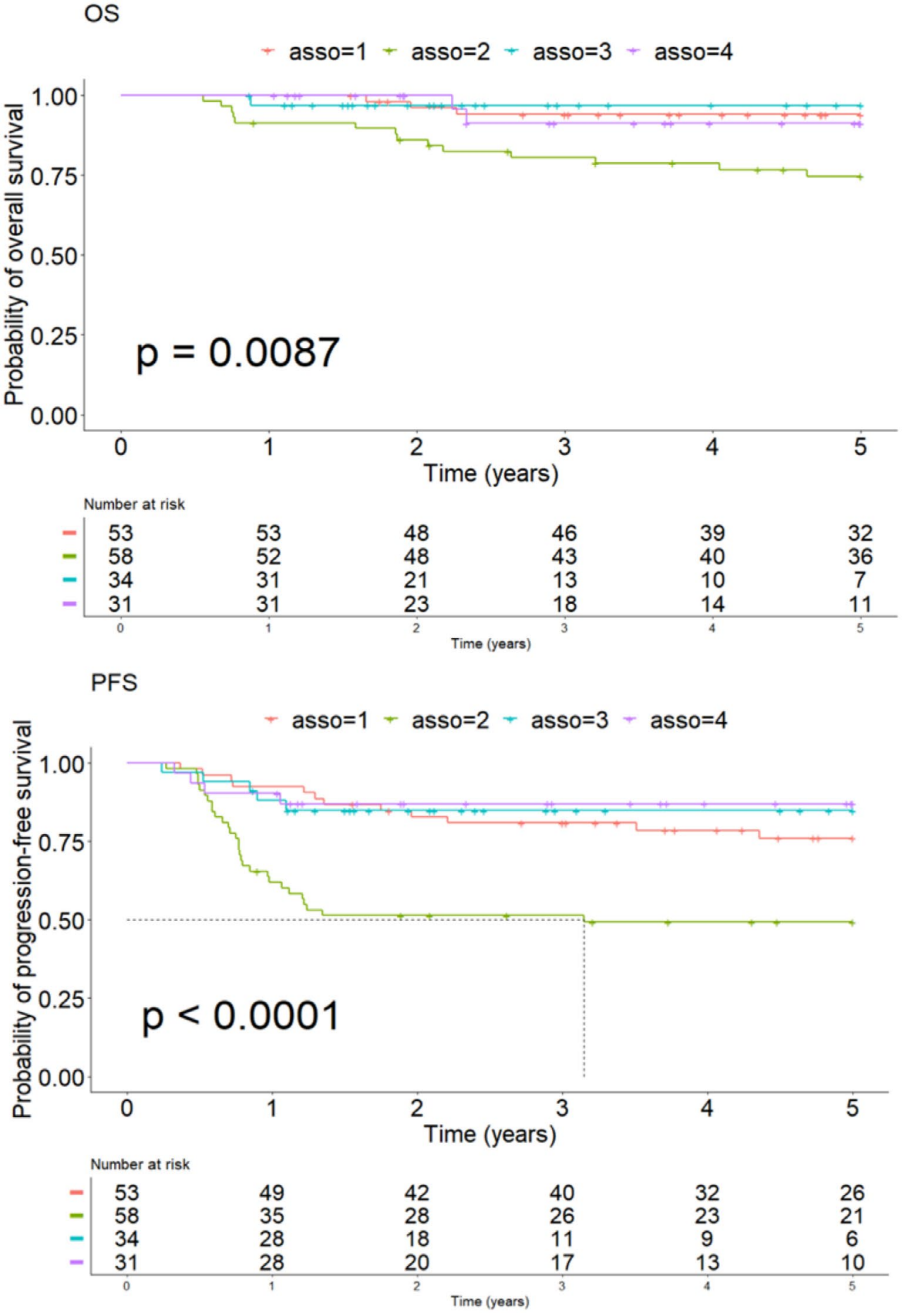
Survival analysis KM curves for OS and PFS according to the combination of treatment regimen + medEdgeD value.

Kaplan–Meier survival curves for OS and PFS are available in Figure 4 and showed a significantly worse prognosis in OS or PFS for patients with high baseline medEdgeD treated by ABVD (Group 2) compared to the others, notably patients treated by the same chemotherapy but with a low baseline medEdgeD value (Group 1).

### 3.8 Subgroup Analysis

**FIGURE 5:**
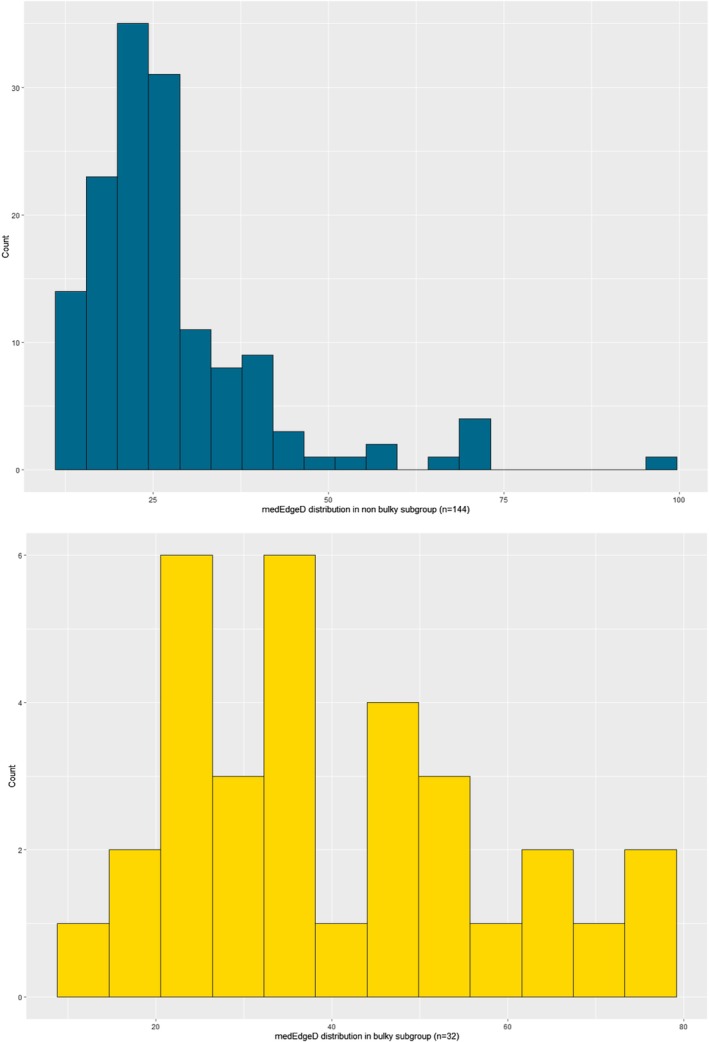
Graphical representation of the distribution of medEdgeD according to the bulky criteria.

**FIGURE 6:**
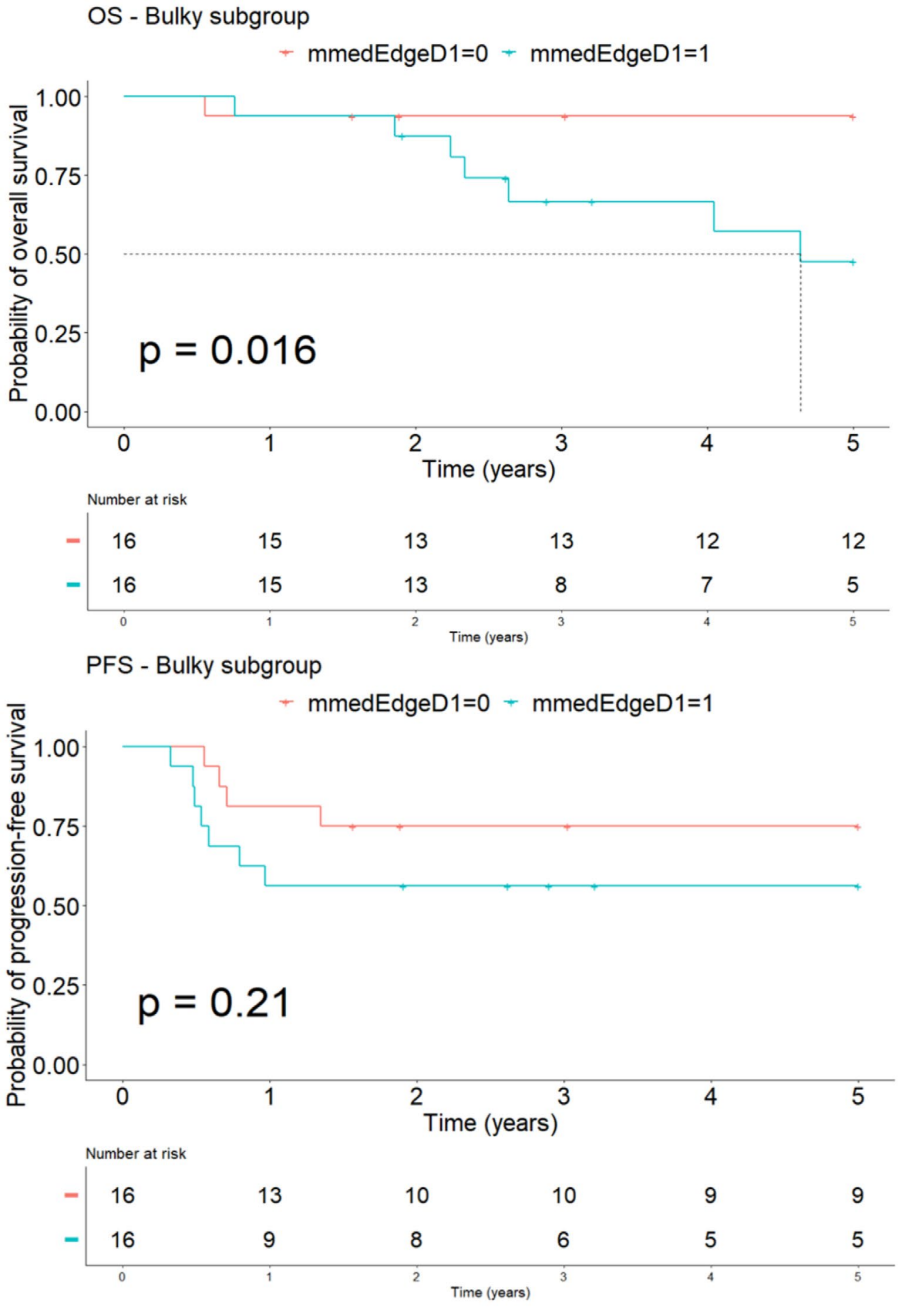
Survival analysis curves for OS and PFS according to the baseline value of medEdgeD in the bulky subgroup (*n* = 32).

As medEdgeD, reflecting the massiveness of a tumor can be linked to the bulky criteria, a subgroup analysis considering that criteria was performed. Figure 5 shown that the distribution of medEdgeD was significantly different between the two groups, bulky negative or bulky positive, with a higher median value in the bulky subgroup (34.3 vs. 24.3 mm; *p* < 0.0001) (Table 8). Furthermore, in the bulky positive subgroup, the Kaplan–Meier survival curve for OS censored at 5 years shows a significantly different survival, with a worse OS for patients with a high baseline medEdgeD than for patients with a low baseline medEdgeD, and only a tendency was highlighted for PFS analysis (Figure 6).

**TABLE 8:** Student's test for comparison of medEdgeD according to the bulky criteria.

Characteristic	*N*	Bulky, *N* = 32	Nonbulky, *N* = 144	*p*
MedEdgeD	176			**< 0.0001**
Mean ± SD		40.07 ± 17.10	27.59 ± 13.28	
Median (25%; 75%)		**34.31** (26.21; 51.99)	**24.34** (19.90; 30.09)	
Minimum; maximum		12.68; 77.20	11.99; 96.18	

*Note:* Values displayed in bold represent statistically significant results (*p* < 0.05).

**FIGURE 7:**
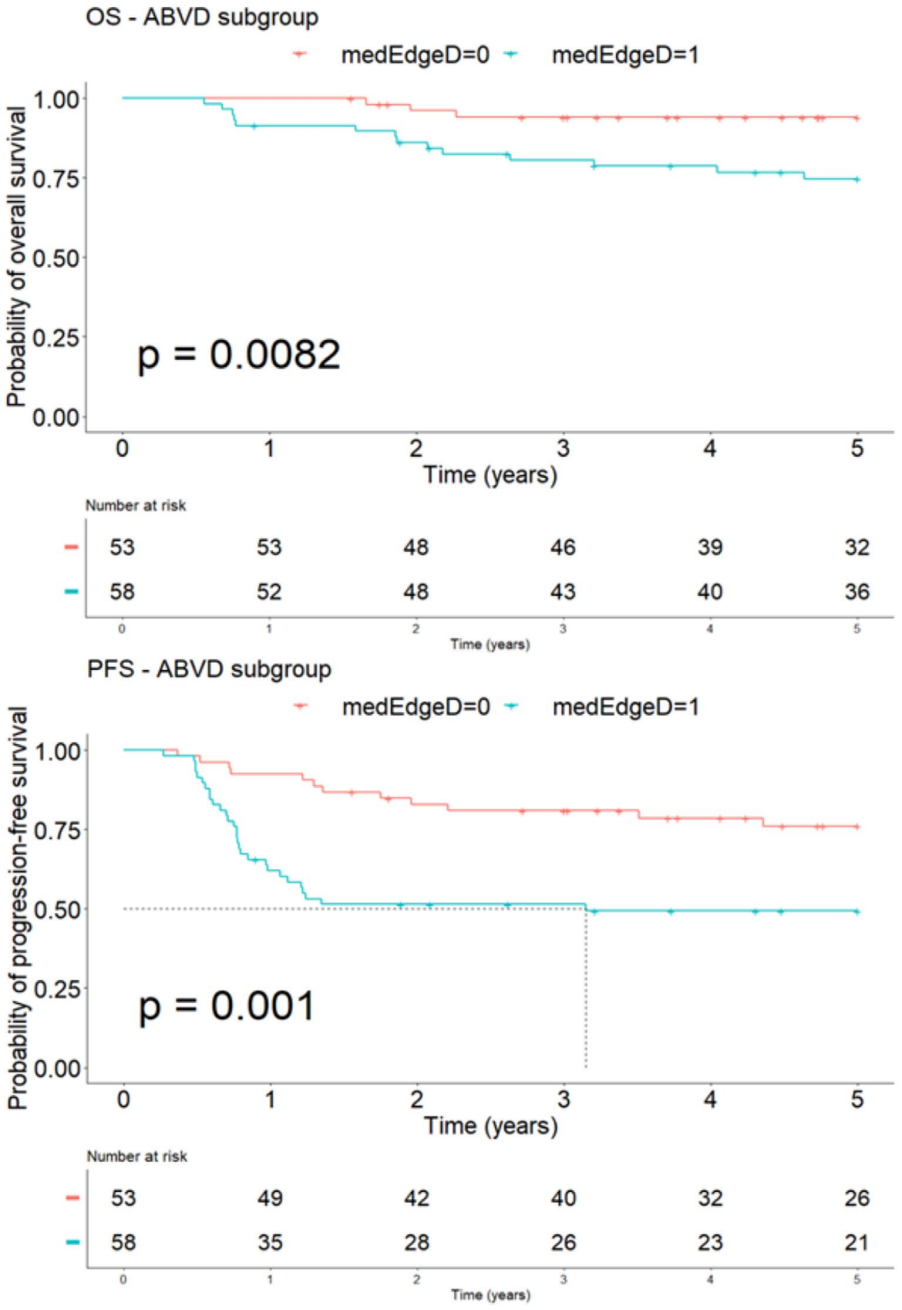
Survival analysis curves for OS and PFS according to the baseline value of medEdgeD in ABVD sub‐group.

**FIGURE 8:**
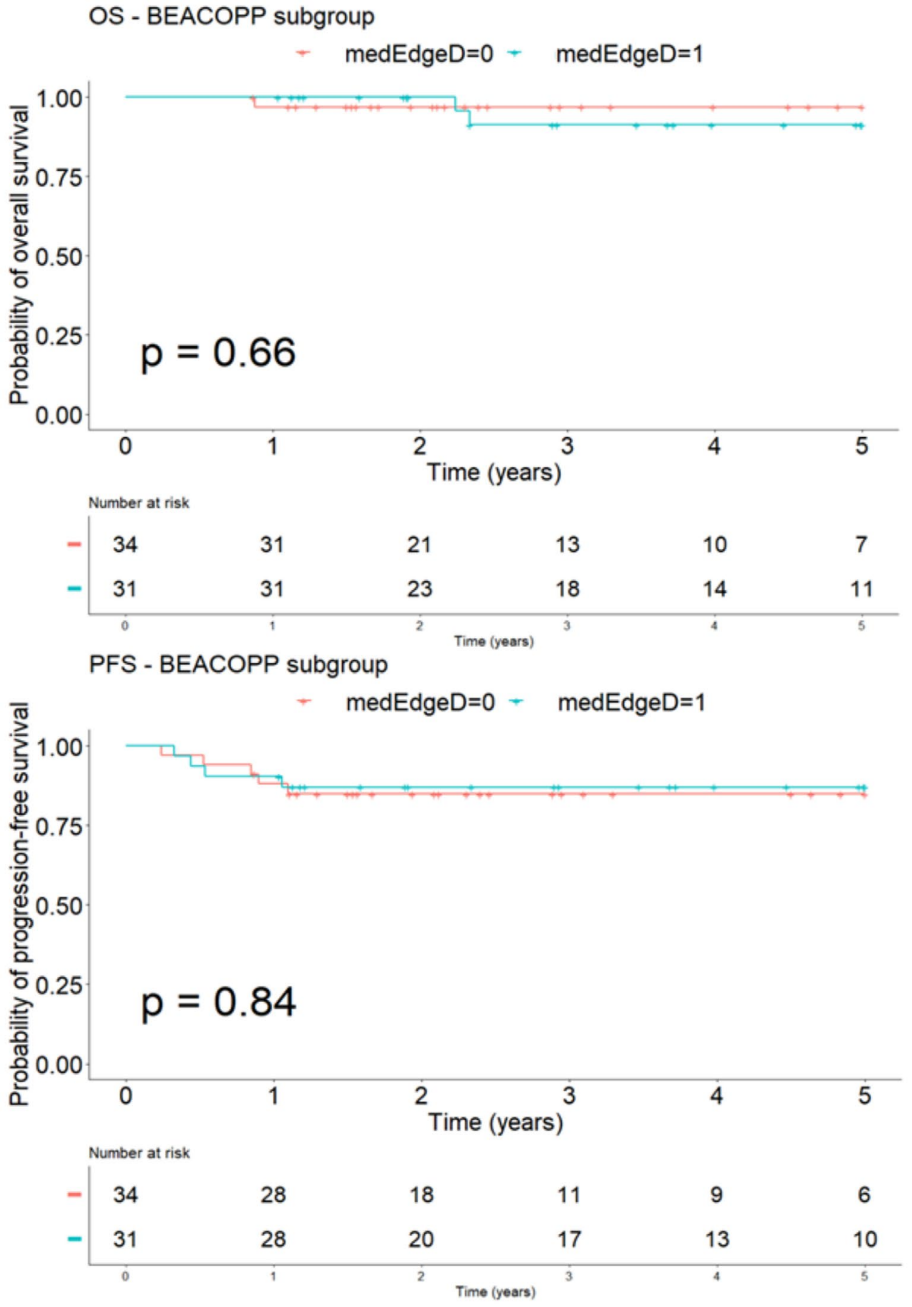
Survival analysis curves for OS and PFS according to the baseline value of medEdgeD in BEACOPP sub‐group.

Consistently, by separating patients according to the treatment regimen, we also showed that medEdgeD had a statistically significant impact on OS and PFS in the ABVD subgroup, not found in the BEACOPP subgroup (Figures 7 and 8).

## 4 Discussion

In the present study, we explored the prognostic value of different ^18^F‐FDG PET/CT‐derived quantitative parameters in a large population of 176 newly diagnosed HL patients. The 12 PET parameters studied explored different aspects of the malignancy: the tumor burden (with TMTV, TMTS, and also with TLG), the tumor activity (SUVmax, SUVmean, and partly with TLG), the tumor dispersion (*D*_max_ and TumBB), and the tumor massiveness‐fragmentation (itErosion, TVSR, medPCD, and medEdgeD), and considered the disease as a whole in order to be able to characterize all locations.

Among the four clusters on PET parameters describing activity, tumor burden, massiveness‐fragmentation, and dispersion, we found that the massiveness‐fragmentation parameters, including medPCD, medEdgeD, itErosion, and TVSR had the highest prognostic value.

These findings are consistent with other studies [16, 22, 24, 25], indicating that PET radiomic features can help with prognostic evaluation in HL patients. To the best of our knowledge, our study is the first one to demonstrate the independent prognostic value of baseline massiveness TEP parameters in advanced‐stage HL, notably the prognostic role of high baseline medEdgeD value.

The primary strength of our study is the novel finding that a high value of medEdgeD at baseline imaging allows the identification of patients presenting a high risk of treatment failure. Furthermore, in the multivariate analysis involving medEdgeD and conventional clinical factors such as the Hasenclever‐index (IPS) score, ECOG performance status, B signs, first‐line treatment, and bulky disease criteria, the massiveness parameter medEdgeD remained a baseline prognostic factor for both OS and PFS prediction.

Moreover, in the bulky sub‐group, high baseline medEdgeD enabled us to individualize a subset of patients with a significantly worse outcome for both PFS and OS compared to the others, indicating that this quantitative parameter has an added prognostic value with respect to the classical bulk criteria. Tumor bulk has been studied for years and has been linked to a bad prognosis. However, its strict definition is still uncertain and varies among studies [26]. Historically, it was defined by the EORTC as in our study, based on the mediastinal mass ratio (maximum width of mass/maximum intra‐thoracic diameter) measured > 0.35 at T5–T6 [27], mostly on a conventional upright chest x‐ray. With CT scan development, various cutoff values were presented (for example: > 10 cm nodal mass) [28] and more recently, TEP parameters such as TMTV or TLG have been proposed to represent bulky disease. MedEdgeD may provide insights into the spatial and structural aspects of the tumor that are not captured by traditional bulk criteria.

MedEdgeD was able to discriminate the prognosis of patients treated by ABVD. Among all patients, the worst prognosis was observed for patients treated with ABVD and having a high MedEdgeD value. Keeping in mind that the main goal is to limit intensive treatment (chemotherapy or radiotherapy) to a subset of patients who might benefit from an aggressive approach to maintain response rate and to minimize the treatment‐related toxicities in the whole population, these patients could potentially benefit from more intense treatment with BEACOPP instead of ABVD, although this needs to be evaluated in a prospective study.

The correlation between these PET parameters and survival can provide insights into the biology of Hodgkin lymphoma. Researchers may investigate the underlying mechanisms that drive the relationship between tumor massiveness and patient outcomes, potentially leading to the discovery and development of targeted therapeutic targets. Our cohort, treated between 2006 and 2017, predates the broad use of newer frontline therapies like BV‐AVD and checkpoint inhibitors. Despite this, medEdgeD remains a relevant prognostic marker because it reflects tumor massiveness, a fundamental biological characteristic likely consistent across treatments. Prospective studies are needed to confirm medEdgeD's predictive value in patients receiving these modern therapies.

On the contrary, we did not retain a significant prognostic value of tumor burden parameters such as TMTV [15, 22, 23, 29] or dissemination parameters such as *D*_max_ [30], capturing the spread of the disease, for OS or even PFS. Only a tendency was shown for activity parameters, in particular with the SUVmean for OS prediction. This highlights the variability in research findings within the field of oncology, and it's not uncommon for different studies to produce differing results. Many sources can explain these differences, such as the sample size and the image segmentation method using a fixed threshold.

Regarding the survival, we chose to make an emphasis on OS due to the higher survival rate of HL compared to other lymphomas [8], with an overall rate estimated around 90% [2], limiting the number of events demonstrated in a single centre.

All images were acquired in a single centre using the same acquisition and reconstruction protocols in order to maximize repeatability and robustness. We used an automatic segmentation method given the fact that HL can be highly heterogeneous in shape, size, or location. PET parameters extraction guided by automatic methods can consistently reduce analysis to less than 5 min per patient compared to a fully manual workflow that can be difficult and time‐consuming, particularly in high disease burden, taking the physician up to 30 minu to extract imaging metrics. In addition, the use of a fixed absolute threshold (SUV4.0), as opposed to manual segmentation, results in better repeatability [31] but remains controversial due to the lack of consensus regarding segmentation methods and its inner limitations, in particular the exclusion of tumors with low FDG uptake, which are rare in baseline PET for LH. The variability of target volume delineation protocols, the lack of validation cohorts, and the lack of methodological harmonization are factors that limit the diffusion of this type of approach in clinical routine [32]. Recent advances in medical imaging software and automated segmentation enable rapid extraction of radiomic parameters, typically within minutes. Although manual verification remains necessary to exclude nonpathological uptake, emerging artificial intelligence and deep learning tools show great promise to automate this process fully, providing near real‐time extraction of prognostic biomarkers. These developments will support the integration of medEdgeD into routine clinical workflows, facilitating personalized patient management [33].

It should be noted that most of the PET/CT‐derived parameters analyzed in this study are geometrical parameters, describing shape and analyzed for the vast majority on PET/CT systems utilizing the same technology. Contrary to the majority of radiomic textural features, these parameters are robust and less sensitive to differences in PET/CT devices or even reconstruction algorithms [34, 35, 36]. Therefore, harmonization of data extracted from different PET/CT scans is unnecessary given the nature of the parameters explored. Furthermore, the PET/CT parameters examined in this study are easily understandable from a biological point of view and calculated directly from baseline PET/CT without the need for any additional cost or dedicated imaging acquisition.

The cohort was limited to advance‐stage IIB bulky‐III and IV HL patients as classified by the GHSG. It is unknown how this approach would perform in low‐burden disease and require separate investigation. Furthermore, these results must be evaluated in patients treated with new treatment regimen combinations, such as brentuximab vedotin (BV) or immune checkpoint inhibitors (CPI) like pembrolizumab.

Our study has some limitations, as its retrospective nature. Therefore, further research in larger multi‐centre studies is suggested to determine the role of quantitative PET in predicting HL outcomes before their implementation as clinical tools. However, the possible integration of medEdgeD into clinical practice highlights the importance of multimodal approaches in hematology. Combining traditional clinical criteria with advanced imaging parameters like medEdgeD can lead to more comprehensive and accurate prognostication. In our study, medEdgeD, a simple quantitative PET parameter, appeared to be an interesting surrogate marker for bulky disease, being more efficient in the selection of patients, more reproducible given the uncertainty in bulk criteria definition, and associated with significantly worse outcome.

## 5 Conclusion

The results of this large monocentric retrospective study highlight the independent prognostic value of massiveness TEP parameters for OS and PFS, notably medEdgeD in patients with advanced‐stage Hodgkin Lymphoma treated with standard chemotherapy, especially ABVD, and are promising in standardizing and improving lymphoma patient care.

## Author Contributions

**S. Draye‐Carbonnier:** investigation, writing – review and editing, writing – original draft. **S.‐D. Mihailescu:** methodology, formal analysis. **P. Pinochet:** formal analysis. **E. Texte:** formal analysis. **A. Stamatoullas‐Bastard:** data curation, supervision. **P. Vera:** supervision. **S. Becker:** data curation. **P. Decazes:** conceptualization, investigation, writing – review and editing, writing – original draft, methodology.

## Funding

The authors have nothing to report.

## Conflicts of Interest

The authors declare no conflicts of interest.

## Supporting information


**Figure S1:** cam471462‐sup‐0001‐FigureS1.pdf.

## Data Availability

The datasets generated for this study are available on request to the corresponding author.
