# Supplementary material for: Prognostic Value of Massiveness Parameters Measured on Baseline FDG PET in Advanced‐Stage Hodgkin Lymphoma
Source: Cancer Med. 2025 Dec 13;14(24):e71462. doi: 10.1002/cam4.71462 (PMC12701620; doi:10.1002/cam4.71462)

Figure S1: *Representation of the twelve different PET parameters measured by the software Oncometer3D and analyzing burden, activity, dispersion, fragmentation and massiveness of the lymphoma. For graphical purpose, a planar representation of these 3D parameters is shown. In Decazes et al, EJNMMI Research, 2020.*

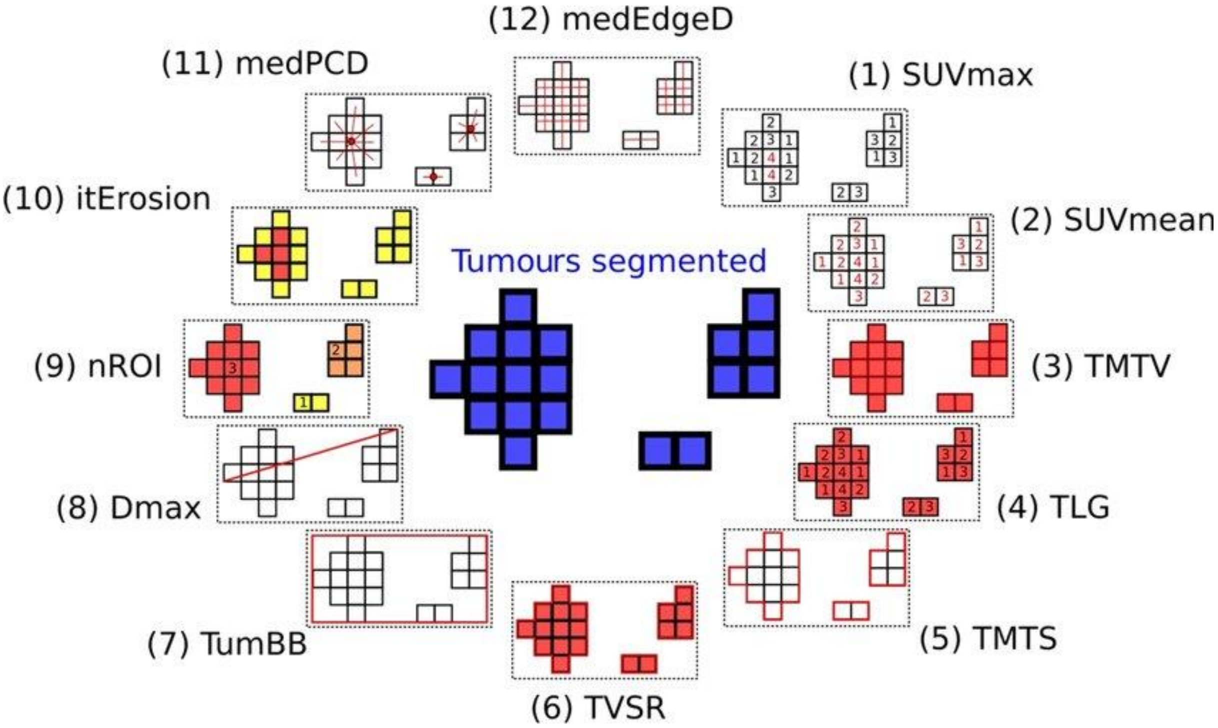

Supplement: Supplementary file 1 — Figure S1: cam471462‐sup‐0001‐FigureS1.pdf. [file CAM4-14-e71462-s001.pdf]
